# Structural gradients in excess mortality during and after the COVID-19 pandemic: economic development, preparedness, and cross-national differences, 2020–2024

**DOI:** 10.7189/jogh.16.04246

**Published:** 2026-07-31

**Authors:** Daeil Jang, Sunhwa Choi, Boseung Choi, Soyoung Kim

**Affiliations:** 1 Innovation Center for Industrial Mathematics, National Institute for Mathematical Sciences, Seongnam, Republic of Korea; 2 Biomedical Mathematics Group, Pioneer Research Center for Mathematical and Computational Sciences, Institute for Basic Science, Daejeon, Republic of Korea; 3 Division of Big Data Science, Korea University, Sejong, Republic of Korea; 4 College of Public Health, The Ohio State University, Columbus, USA

**Keywords:** excess mortality, COVID-19, pandemic preparedness, economic development, structural capacity, Global Health Security Index

## Abstract

**Background:**

Excess mortality has emerged as a comprehensive indicator of overall mortality burden of the COVID-19 pandemic, reflecting both direct and indirect deaths beyond officially reported COVID-19 fatalities. However, the substantial cross-national variation in excess mortality remains incompletely explained, particularly in relation to structural heterogeneity across countries. This study examines whether pandemic mortality differs according to economic development and national preparedness.

**Methods:**

We analysed 59 countries from 2020 to 2024. Excess mortality ratios were estimated based on pre-pandemic mortality trends (2015–2019). Countries were classified as advanced or emerging market economies based on the International Monetary Fund criteria. National preparedness was assessed by the 2021 Global Health Security (GHS) Index. Associations between preparedness and excess mortality were evaluated using regression models and interpreted using SHapley Additive exPlanations (SHAP)-based analysis to quantify domain-level contributions to predict mortality.

**Results:**

Excess mortality peaked globally in 2020 and 2021 and declined thereafter. During the early period of the pandemic, emerging market economies experienced consistently higher total and non-COVID (excluding officially reported COVID-19 deaths) excess mortality than did advanced economies. Using the pooled analyses during the early pandemic period (2020–2021), higher overall GHS Index scores were significantly associated with lower excess mortality. However, this association weakened after stratification by economic development stage, indicating that the pooled associations were largely driven by structural differences between economic groups. Early detection and risk environment as preparedness domains were strongly associated with reduced excess mortality.

**Conclusions:**

Cross-national differences in pandemic mortality were primarily shaped by structural capacity gradients rather than preparedness scores. Therefore, preparedness indices might function better as indicators of broader structural capacity than predictors of mortality outcomes in comparable economic contexts. Strengthening pandemic resilience should prioritise surveillance capacity and institutional robustness to enhance baseline preparedness capacity.

The COVID-19 pandemic has functioned as a large-scale systemic stress test, exposing profound systemic disparities in mortality outcomes worldwide. Although some countries have mitigated fatalities through timely public health interventions and a resilient healthcare infrastructure, others have faced substantial and prolonged increases in mortality. These stark cross-national differences may not be solely attributable to short-term policy responses or viral dynamics; rather, they indicate that fundamental structural factors shape national vulnerability. The crisis underscored deep-seated inequalities in states’ capacities to detect, absorb, and mitigate large-scale systemic shocks [1–3].

A key challenge in assessing cross-country variations in the impact of the COVID-19 pandemic is the use of comparable mortality indicators. Officially reported COVID-19 deaths are widely recognised as underestimating the true mortality burden due to cross-national differences in testing capacity, diagnostic practices, and cause-of-death attribution [4–6]. Therefore, excess mortality has emerged as a more reliable measure of the overall impact of a pandemic. Excess mortality is defined as the difference between the observed deaths and the number of deaths expected based on historical baseline trends. Comparing the observed deaths with pre-pandemic baselines, it captured both deaths directly caused by SARS-CoV-2 and indirect deaths resulting from healthcare disruptions and broader systemic strains [7,8]. To further disentangle these components, separating excess mortality, excluding officially reported COVID-19 deaths, allows for a more focused assessment of indirect mortality burdens. This approach provides additional insights into the extent to which excess mortality reflects healthcare disruptions, delayed treatment, and broader systemic strains rather than direct viral mortality alone.

The substantial variation in excess mortality across countries has intensified the debate on the role of national preparedness frameworks in shaping pandemic outcomes. The Global Health Security (GHS) Index is a widely used benchmark for evaluating national capacities for prevention, detection, and response [9–11]. During the early pandemic, higher preparedness scores were expected to correspond to lower mortality rates. However, the emergence of the ‘GHS paradox’, where several highly ranked countries experienced severe outbreaks while some lower-ranked countries reported comparatively lower mortality, challenged this expectation. These observations raise questions about whether conventional preparedness metrics adequately reflect the complex determinants of pandemic performance or whether they overlook the broader sociostructural contexts in which these capacities operate [12–14].

Rather than indicating a fundamental failure of preparedness metrics, this paradox might reflect the structural heterogeneity across countries. Nations substantially differ in economic development, demographic structure, governance capacity, and baseline health-system resilience. Economic development was used as the primary stratification factor because it provides an integrative proxy for broader structural capacity, including institutional stability, governance effectiveness, socioeconomic resources, and health system conditions. Although factors (population size, regional location, and specific healthcare capacity indicators) might influence pandemic outcomes, they reflect more limited dimensions of the national context and are, thereby, not used as the main stratification criteria. Analysing such heterogeneous contexts together, the associations between preparedness indicators and pandemic outcomes might be distorted by aggregation bias [15,16]. Therefore, structural differences between countries at different stages of economic development might generate cross-national correlations that do not reflect relationships within comparable contexts [17–20]. Despite growing research on pandemic preparedness, how preparedness-mortality relationships vary across structural capacity levels remains insufficiently assessed.

Previous studies assessing the relationship between the GHS Index and COVID-19 outcomes have reported inconsistent results. Many early analyses were limited to the first year of the pandemic and did not reflect the mortality dynamics under prolonged systemic stress [21,22]. Moreover, most studies have relied on composite preparedness scores and assumed linear relationships, which might obscure the influence of individual preparedness domains. Such approaches might overlook the possibility of threshold effects [23,24].

To address these critical gaps, this study examined the cross-national patterns of excess mortality in 59 countries over the longitudinal course of the COVID-19 pandemic (2020–2024). Excess mortality served as the primary outcome measure, analysed both in total and net officially reported COVID-19 deaths, to isolate the burden of indirect mortality. Countries were stratified by economic development status according to the International Monetary Fund classification to account for differences in structural capacity between advanced and emerging market economies [25,26]. Using multivariable regression and explainable machine learning using SHapley Additive exPlanations (SHAP) to uncover the nonlinear contributions of specific preparedness domains, we examined how domains of health security preparedness relate to excess mortality across different structural contexts. This approach allowed us to identify the preparedness domains that were most closely associated with mortality in different structural contexts.

## METHODS

### Study design

This country-level observational study assessed the relationship between national health security preparedness and excess mortality during the COVID-19 pandemic, from 2020 to 2024. The analysis included 59 countries with complete monthly mortality reports and available data. Countries were included if continuous monthly all-cause mortality data were available for both the pre-pandemic baseline and pandemic periods to ensure temporal comparability. Countries were classified as advanced or emerging market economies according to the IMF criteria, and economic development served as a primary stratification factor to account for the differences in structural capacity.

The analysis was performed in three sequential steps. First, we assessed the temporal patterns of excess mortality and compared the distributions across the pandemic years between advanced and emerging market economies. Second, we evaluated the association between preparedness and excess mortality across the full study period (2020–2024) using linear regression models, with and without stratification by economic development. Year-specific analyses were performed to assess the temporal robustness of these associations across the different pandemic phases. Third, we investigated which preparedness domains were most strongly associated with excess mortality using a multivariate regression model and interpreted the relative contributions of the preparedness domains separately for each economic group using the SHAP. This approach allows a detailed interpretation of domain-level contributions under conditions in which cross-country comparability is maximised.

### Data

#### All-cause mortality and GHS Index

All-cause mortality data were collected from the World Mortality Data set compiled by Karlinsky and Kobak [7], which included mortality reports from 127 countries. To ensure completeness and temporal consistency, we restricted the sample to countries with continuous monthly mortality data without missing values across both the pre-pandemic baseline and study periods (January 2015–December 2024).

By applying this criterion, 63 countries were retained and matched with available GHS Index data, resulting in a final analytical sample of 59 countries. Countries lacking complete mortality data or corresponding GHS Index values were excluded to avoid bias due to incomplete time series or inconsistent reporting.

A list of the included countries and their classifications by economic development status is presented in Table S1 in the **Online Supplementary Document**. To assess the representativeness of the analytical sample, we compare the GDP distribution of the included countries with that of a broader set of countries. This comparison shows that the analytical sample was concentrated in the upper range of the global GDP distribution (Figure S1 in the **Online Supplementary Document**), reflecting the requirement for complete mortality reporting.

#### National health security preparedness

National pandemic preparedness was measured by the GHS Index 2021 [27], which includes an overall preparedness and six domain-specific scores. We refer to these domains using abbreviated labels: Prevention, Early detection, Response, Health system, Compliance, and Risk environment, corresponding to their full titles defined in the GHS Index. Higher scores indicate greater preparedness.

The GHS Index 2021 is considered a structural indicator of national preparedness, reflecting baseline capacity rather than responses that vary over time during the pandemic. This allows interpreting preparedness as a stable, pre-existing structural characteristic rather than a short-term policy response. The full names and abbreviations of the six domains and the country-specific overall and domain-level GHS Index scores are presented in Tables S2 and S3 in the **Online Supplementary Document**.

### Statistical analysis

We first calculated the excess mortality ratio for each country, which served as the primary outcome variable throughout the analysis. Using these ratios as standardised measures of the pandemic-related mortality burden, we evaluated annual trends (2020–2024) using boxplots stratified by economic development status. The differences between advanced and emerging market economies were determined each year to identify the periods with the greatest divergence in mortality outcomes. Year-specific differences in the mean excess mortality ratios were evaluated using Welch’s *t* tests. The same stratified approach was applied to the excess mortality ratios, which excluded officially reported COVID-19 deaths. These analyses established temporally informed subsequent regression analyses performed across the entire study period.

#### Excess mortality ratio estimation

The expected mortality in the absence of the pandemic was estimated using annual all-cause mortality data from the pre-pandemic period (2015–2019). For each country, a linear trend was fitted to this baseline period and used for predicting the expected deaths for each year within 2020–2024.

Excess mortality ratios were calculated by subtracting expected deaths from observed deaths and then dividing by expected deaths; zero indicates baseline mortality, and positive values indicate excess mortality. To assess mortality patterns beyond confirmed COVID-19 deaths, we calculated excess mortality ratios, excluding officially reported COVID-19 fatalities, by subtracting confirmed COVID-19 deaths from observed all-cause deaths before the calculation.

#### Linear regression analysis

The association between preparedness and excess mortality was assessed by linear regression models. The models were estimated for each year to evaluate the temporal robustness of these associations across the different pandemic phases.

First, we estimated a pooled model including all countries:

EMR*_i,t_* = *α + β*_t_⋅GHS*_i_* + ε*_i,t_*

where EMR*_it_* denotes the excess mortality ratio for country *i* in year *t* (2020–2024) and GHS*_i_* is the GHS Index overall score.

Second, to assess whether this association differed by economic development stage, we estimated separate regression models for advanced and emerging market economies.



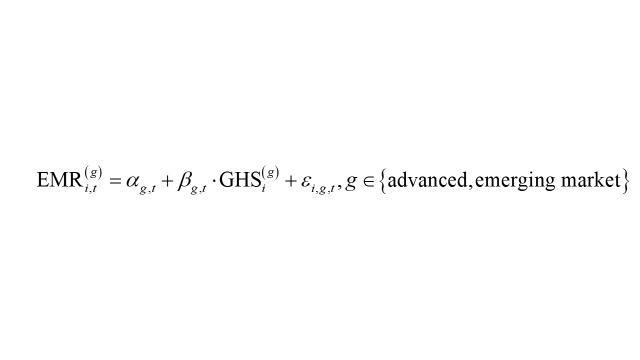



These stratified models allowed us to evaluate within-group associations and distinguish cross-country structural differences from variation within economically similar groups.

To determine the aspects of preparedness that were most closely linked to excess mortality, we estimated a multiple linear regression model that included all six GHS domain scores and economic development status as explanatory variables.



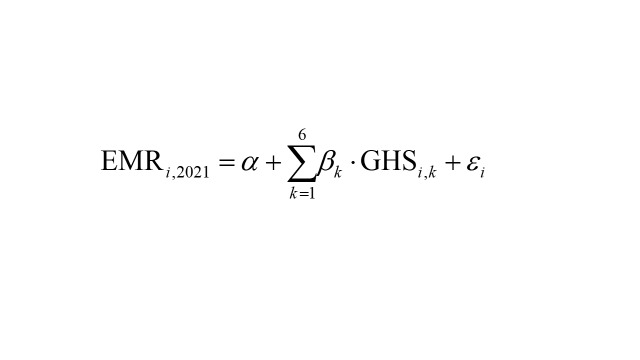



Where GHS*_i,k_* represents the score for domain *k* in country *i*.

To interpret the domain-level contributions within each economic group, we applied SHAP using a linear explainer consistent with the fitted regression model. The SHA*P* values quantify the contribution of each preparedness domain to the predicted excess mortality for a given country, indicating both the direction and magnitude of its effect. The SHA*P* values were computed for each country and summarised separately for advanced and emerging market economies. Representative SHAP decision plots were generated for each group to illustrate how preparedness domains cumulatively contributed to the predicted excess mortality. In these plots, bars extending left indicate a decrease in the predicted excess mortality ratio, whereas bars extending right indicate an increase.

All analyses were performed using Python 3.11, with Pandas [28] for data processing, *statsmodels* [29] for regression analysis, and the SHAP library for model interpretation.

## RESULTS

### Annual excess mortality by economic development stage

**Figure 1:**
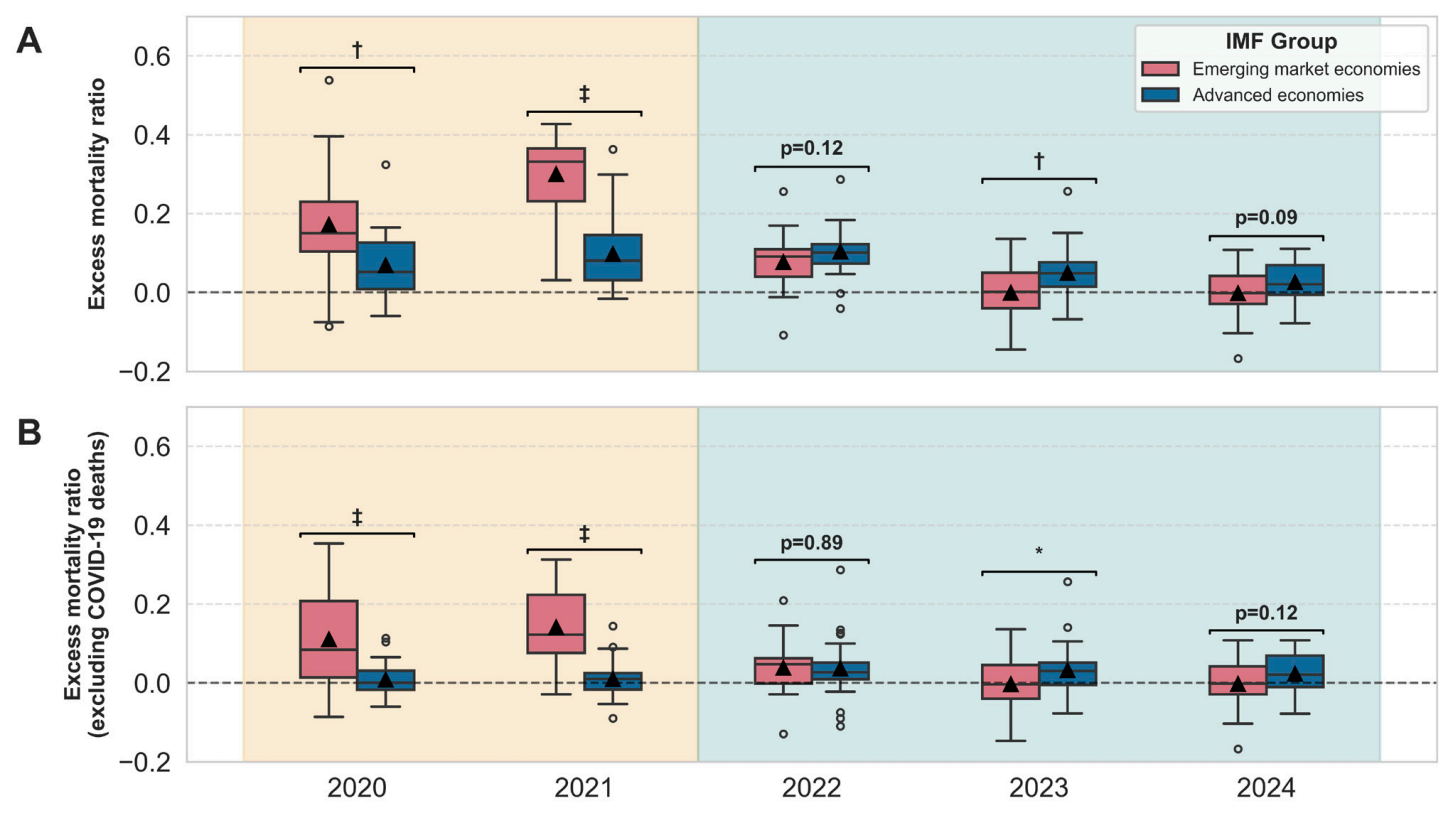
Annual excess mortality ratios by economic development stage, 2020–2024. Panel A. Total excess mortality ratios across pandemic years for advanced and emerging market economies classified according to International Monetary Fund (IMF) criteria. Panel B. Excess mortality ratios excluding officially reported COVID-19 deaths. Boxplots summarise country-level distributions for each year, with medians, interquartile ranges, and outliers displayed. Mean differences between groups were tested for each year using Welch’s *t* tests. *P < 0.05. †*P* < 0.01. ‡*P* < 0.001. IMF – International Monetary Fund.

Excess mortality ratios were the highest during the early phase of the COVID-19 pandemic and declined substantially in later years (**Figure 1**, Panel A). Between 2020 and 2021, emerging market economies consistently exhibited higher excess mortality than did advanced economies, indicating a pronounced structural disparity in pandemic mortality during the acute phase of the crisis. The difference between the two economic groups is most pronounced in 2021 and reaches statistical significance. From 2022 onward, the excess mortality ratios markedly declined in both groups and gradually converged toward baseline levels, without substantial or consistent differences found between advanced and emerging market economies in subsequent years.

Recalculating excess mortality, excluding officially reported COVID-19 deaths (**Figure 1**, Panel B), a more asymmetric pattern emerged between economic groups. Non-COVID excess mortality remained close to zero in advanced economies throughout the study period, whereas emerging market economies exhibited elevated levels during the early years of the pandemic. In 2020 and 2021, the median non-COVID excess mortality ratio in emerging market economies is approximately 0.10, corresponding to a 10% increase in mortality relative to baseline levels. This pattern suggests that indirect mortality associated with healthcare disruption and systemic strain was disproportionately concentrated in emerging market economies during the early phases of the pandemic. From 2022 onward, the excess mortality declined in emerging market economies and remained near zero in both groups.

### Distributions of GHS Index scores by economic development stage

The GHS Index scores systematically differed between advanced and emerging market economies. Across all indicators, advanced economies consistently exhibit higher preparedness scores than do emerging market economies, both for the overall index and for all six domain-specific components (**Table 1**). For most indicators, these differences were statistically significant, indicating substantial disparities in baseline preparedness capacity according to economic development stage.

**Table 1:** Comparison of GHS Index scores by economic development stage

Section	Advanced, x̄ (SD)*	Emerging market, x̄ (SD)*	Mean difference†	*P*-value
Overall	57.90 (9.36)	47.37 (9.16)	10.52 (95% CI = 5.61–15.45)	0.0001
Prevention	50.30 (11.64)	42.60 (14.07)	7.70 (95% CI = 0.68–14.72)	0.0324
Early detection	53.97 (17.48)	43.16 (18.35)	10.81 (95% CI = 1.22–20.40)	0.0280
Response	51.07 (10.49)	42.03 (11.55)	9.04 (95% CI = 3.11–14.98)	0.0036
Health system	54.85 (12.27)	43.09 (13.05)	11.75 (95% CI = 4.96–18.54)	0.0011
Compliance	61.17 (13.03)	53.75 (10.79)	7.42 (95% CI = 1.18–13.66)	0.0207
Risk environment	75.99 (7.08)	59.62 (7.04)	16.37 (95% CI = 12.61–20.13)	<0.0001

CI – confidence interval, GHS – Global Health Security, SD – standard deviation, x̄ – mean

*Values are presented as mean (standard deviation) for advanced and emerging market economies classified according to International Monetary Fund criteria.

†Mean differences are shown as advanced minus emerging market economies, with corresponding 95% confidence intervals.

Among the six domains, the largest difference between the two groups was found in the Risk environment domain. Advanced economies show markedly higher median scores, whereas emerging market economies show substantially lower scores in this domain. Differences were also evident in other preparedness domains, including early detection, response, and health system; however, the magnitude of the separation was smaller than that observed in the risk environment domain.

### Association between GHS Index and excess mortality

**Figure 2:**
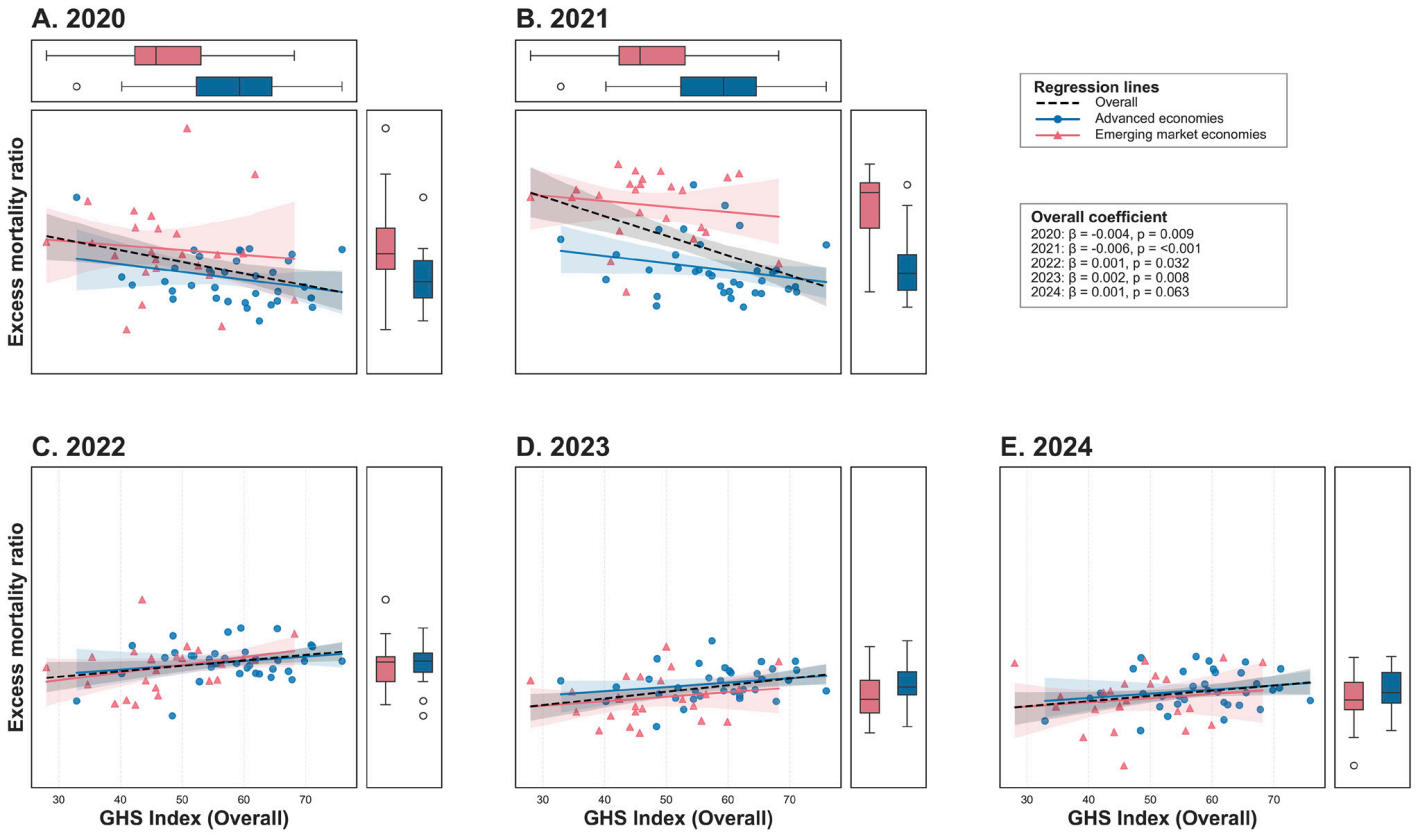
Association between Global Health Security (GHS) Index scores and excess mortality ratios across pandemic years. Panels A–E. The year-specific analyses for 2020–2024. In each panel, excess mortality ratios are plotted against the GHS index scores, with points representing countries and colours indicating economic development groups. The dashed black line shows the pooled regression and the solid lines indicate group-specific regressions with 95% confidence intervals. The top and right subplots show the distributions of GHS Index scores and excess mortality ratios by economic group. GHS – Global Health Security.

Year-specific regression analysis was performed to assess the relationship between national preparedness and excess mortality. Using the pooled analyses, higher overall GHS Index scores were significantly associated with lower excess mortality rates in 2020 and 2021. However, this inverse association was not sustained in later years; from 2022 to 2024, the associations showed weak statistical significance; the estimated coefficients were small in magnitude, suggesting limited practical relevance. After stratification by economic development stage, these associations were further attenuated, with neither advanced nor emerging market economies demonstrating statistically significant relationships in most years (**Figure 2**).

### Domain-specific contributions of GHS indicators to excess mortality

To further determine which preparedness domains contributed the most to excess mortality, we focused on 2021 as the most informative period characterised by widespread transmission and sustained systemic stress across countries, providing a more comparable context for cross-country analysis. We estimated a multiple linear regression model including the six GHS domain scores and evaluated their contributions using SHA*P* values. The SHA*P* values quantify the extent to which each domain contributes to increasing or decreasing the predicted excess mortality for a given country, providing an interpretable decomposition of the model output. Consistent with the regression results, only two domains, Early detection and Risk environment, showed statistically significant associations with excess mortality, whereas the remaining domains minimally contributed to the model output.

**Figure 3:**
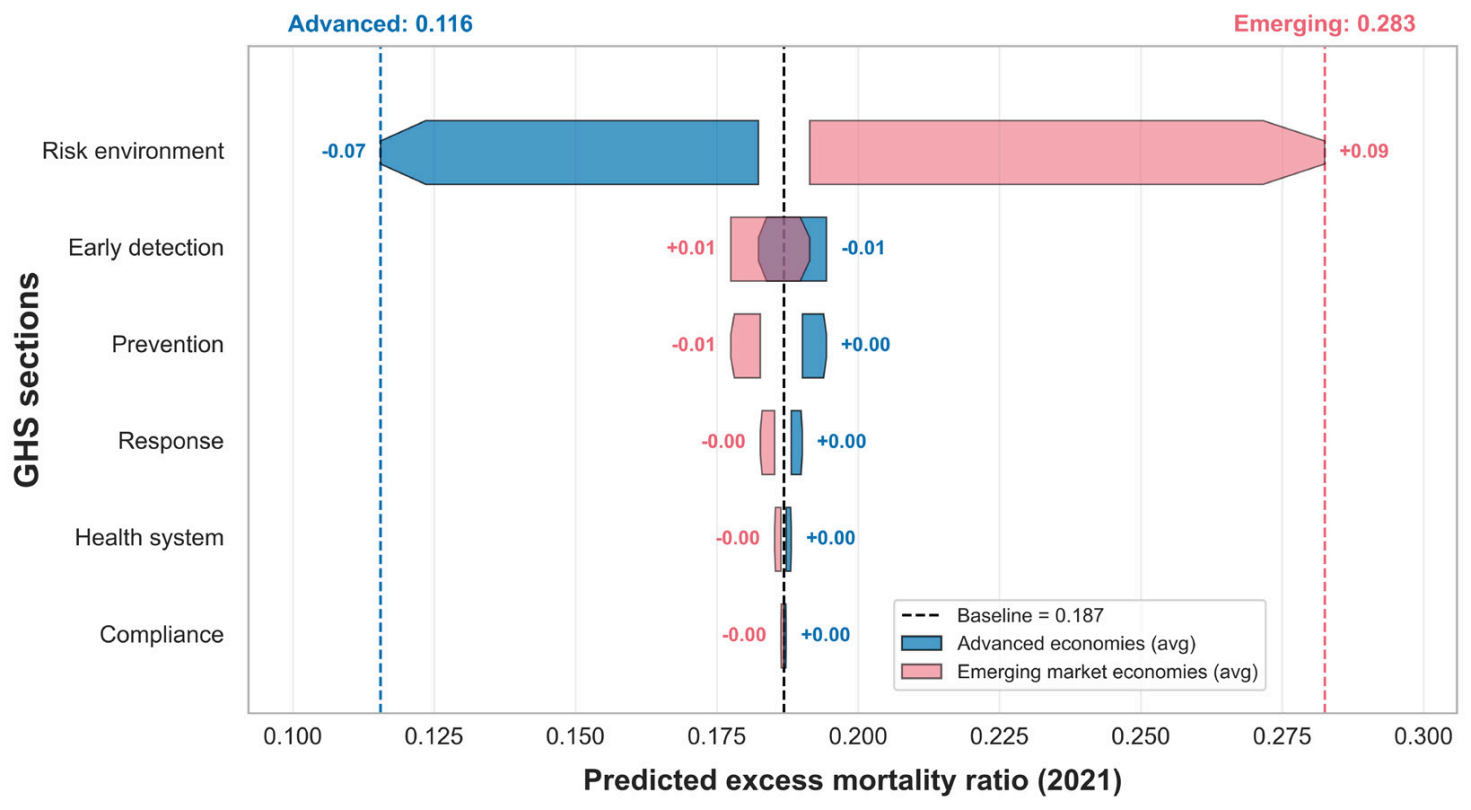
Domain-specific contributions of Global Health Security (GHS) Index indicators to excess mortality in 2021. The figure illustrates SHapley Additive exPlanations (SHAP) contributions derived from a multiple linear regression model including the six domains of the GHS Index. Bars represent the contribution of each domain to predicted excess mortality relative to the model baseline (black dashed line). Positive values indicate increased predicted excess mortality, whereas negative values indicate reduced mortality. Contributions are shown for advanced and emerging market economies according to International Monetary Fund classifications. GHS – Global Health Security.

Across both economic groups, the SHAP results indicated that higher scores in these two domains – early detection and risk environment – contributed to lower predicted excess mortality, whereas lower scores contributed to higher predicted mortality (**Figure 3**). This suggests that variations in these domains played a central role in shaping the differences in predicted mortality across countries under comparable pandemic conditions.

The Risk environment consistently made larger contributions to model predictions than the other preparedness domains in both economic groups. The baseline predicted excess mortality ratio was 0.187. For advanced economies, the domain contributions shifted the prediction downward to 0.116, whereas for emerging market economies, they shifted the prediction upward to 0.283. Although the same domains were influential across groups, the magnitude and direction of their contributions markedly differed between advanced and emerging market economies, reflecting broader structural disparities in preparedness capacity and the national risk context (**Figure 3**).

Detailed regression estimates corresponding to this model are presented in Table S4 in the **Online Supplementary Document**.

## DISCUSSION

This study demonstrated that excess mortality during the COVID-19 pandemic followed a clear structural gradient aligned with national economic development. During the early pandemic years (2020–2021), emerging market economies experienced substantially higher excess mortality than did advanced economies, even after excluding officially reported COVID-19 deaths. This pattern suggests that structural vulnerabilities influence not only direct viral mortality, but also indirect deaths resulting from healthcare disruptions and systemic strain [30–32]. The pandemic acted as a large-scale stress test of health system resilience across countries, exposing underlying inequalities in countries’ capacities to withstand large-scale health shocks.

Pooled analyses generally showed that higher GHS Index scores were associated with lower excess mortality during the early pandemic phase (2020–2021). However, this association weakened after stratification based on economic development. This pattern reflects an aggregation phenomenon in which cross-national structural differences generate significant pooled associations that disappear in more comparable contexts [19,33]. The widely discussed ‘GHS paradox’, where highly ranked countries experienced severe early pandemic outcomes, might reflect structural heterogeneity rather than a failure of preparedness metrics themselves [34].

Focusing on 2021 as the representative period provides an important context for interpreting these findings. By this stage of the pandemic, SARS-CoV-2 transmission had become globally widespread, placing sustained pressure on national health systems [35]. Compared with the staggered outbreak patterns in 2020, this period represents a more synchronised phase of systemic stress. Moreover, vaccination coverage and institutional adaptation remain uneven across countries, allowing the baseline preparedness capacity to manifest mortality outcomes more clearly [36].

At the domain level, early detection capacity and national risk environment were the preparedness components most strongly associated with excess mortality. These domain-level associations were identified based on the 2021 analysis, which provided a representative context for interpreting preparedness effects under sustained systemic stress. These findings emphasise the importance of timely surveillance and favourable governance conditions for mitigating mortality during large-scale biological shocks. Delayed detection of community transmission can rapidly overwhelm clinical capacity, whereas a broader risk environment reflecting governance stability, institutional coordination, and public trust shapes the effectiveness of response measures [37–40].

However, the healthcare system capacity showed a weaker association with excess mortality than expected. Although clinical resources remain essential for treating severe cases, medical capacity alone appears insufficient to compensate for delayed detection or broader systemic vulnerabilities under conditions of sustained pandemic stress [41,42]. This finding, derived from the 2021 analysis, aligns with broader concepts of health system resilience, which emphasises the interaction between surveillance systems, governance structures, and societal trust in determining a system’s ability to absorb shocks.

Our findings are consistent with the possibility that mortality reductions might reflect nonlinear or threshold-like dynamics rather than a strictly linear relationship with preparedness indicators. Improvements in outcomes appear to occur once key preparedness domains reach functional operational capacity rather than through incremental improvements across all indicators. In high-capacity settings, additional investments may produce diminishing returns, whereas in structurally constrained environments, deficiencies in these core domains can substantially amplify mortality risk [43,44].

From a policy perspective, these findings highlight the importance of strengthening the fundamental components of pandemic preparedness. Investments in surveillance systems, early outbreak detection, and coordinated public health governance may be associated with greater mortality reduction than expanding clinical capacity alone [45,46]. Improving rapid detection and institutional coordination might be particularly important for mitigating direct and indirect mortality during future health emergencies [47].

This study has several limitations. First, as an observational analysis, the causal relationships between preparedness indicators and mortality outcomes cannot be definitively established. Second, the use of a single GHS Index measure assumes relative stability in preparedness capacity over time and may not capture dynamic policy adaptations during the pandemic [26]. Third, although excess mortality provides a more standardised measure than reported COVID-19 deaths, cross-national differences in death registration and reporting practices may still influence the estimates. Fourth, although VIF values were below common thresholds and ridge regression showed similar results, correlations among GHS domain scores may still have affected the regression coefficients and SHAP rankings. Therefore, the domain-level results should be interpreted as relative patterns rather than fully independent effects. Finally, the analytical sample was skewed toward higher-income countries, as reflected in its position within the upper range of the global GDP distribution (Figure S1 in the **Online Supplementary Document**), which might limit the generalisability of the findings to lower-income settings.

Taken together, our findings suggest that national preparedness indicators should be interpreted primarily as markers of the broader structural capacity embedded within institutional and socioeconomic contexts. Pandemic mortality reflects the structural gradients shaped by economic development, governance conditions, and surveillance capacity [48]. These relationships were most evident during periods of widespread transmission and systemic stress, underscoring the importance of context in interpreting preparedness–mortality associations. Addressing these structural determinants is essential for strengthening global pandemic resilience and reducing mortality during future public health emergencies [49].

## CONCLUSIONS

This study provides evidence that cross-national disparities in pandemic mortality reflect broad structural gradients in national capacity rather than technical preparedness scores in isolation. Although higher GHS Index scores were associated with reduced excess mortality in pooled analyses during the early pandemic phase, this relationship was attenuated upon stratification by economic development, indicating that preparedness metrics primarily reflect the underlying structural heterogeneities across countries. Domain-level decomposition through explainable machine learning underscores that Early detection and national Risk environment are the most critical determinants of mortality outcomes. These patterns were most evident during periods of widespread transmission and systemic stress. Furthermore, our findings are consistent with a functional threshold interpretation, suggesting that mortality reductions might depend on achieving baseline operational readiness in key domains, rather than on incremental improvements across all indicators. Consequently, strengthening foundational surveillance systems and reinforcing institutional robustness should be prioritised to enhance pandemic resilience across countries and mitigate direct and indirect burdens of future health emergencies.

## Additional material


Online Supplementary Document

## Data Availability

**Data availability:** The data used in this study were obtained from publicly available sources. Additional information regarding the processed data and analytical materials is available from the corresponding author upon reasonable request.
