# Supplementary material for: Structural gradients in excess mortality during and after the COVID-19 pandemic: economic development, preparedness, and cross-national differences, 2020–2024
Source: J Glob Health. 2026 Jul 31;16:04246. doi: 10.7189/jogh.16.04246 (PMC13424747; doi:10.7189/jogh.16.04246)
Supplement: Online Supplementary Document [file jogh-16-04246-s001.pdf]

Supplement to: Jang D, Choi S, Choi B, Kim S. Structural gradients in excess mortality during and after the COVID-19 pandemic: economic development, preparedness, and cross-national differences, 2020–2024. J Glob Health. 2024;16:04246.

Table S1. Countries included in the analysis and economic development classification.

IMF economic development classification	Number of country	Country			
Advanced economy	35	Australia	Austria	Belgium	Canada
		Croatia	Cyprus	Czechia	Denmark
		Estonia	Finland	France	Germany
		Greece	Iceland	Ireland	Israel
		Italy	Japan	Republic of Korea	Latvia
		Lithuania	Luxembourg	Malta	Netherlands
		New Zealand	Norway	Portugal	San Marino
		Singapore	Slovakia	Slovenia	Spain
		Switzerland	United Kingdom	United States	-
Emerging market economy	24	Albania	Armenia	Azerbaijan	Bosnia
		Brunei	Bulgaria	Ecuador	Egypt
		Georgia	Hungary	Kazakhstan	Kyrgyzstan
		Malaysia	Mongolia	Montenegro	North Macedonia
		Oman	Poland	Romania	Russia
		Serbia	South Africa	Thailand	Turkey

Figure S1. Distribution of GDP per capita for the analytical sample relative to all countries.

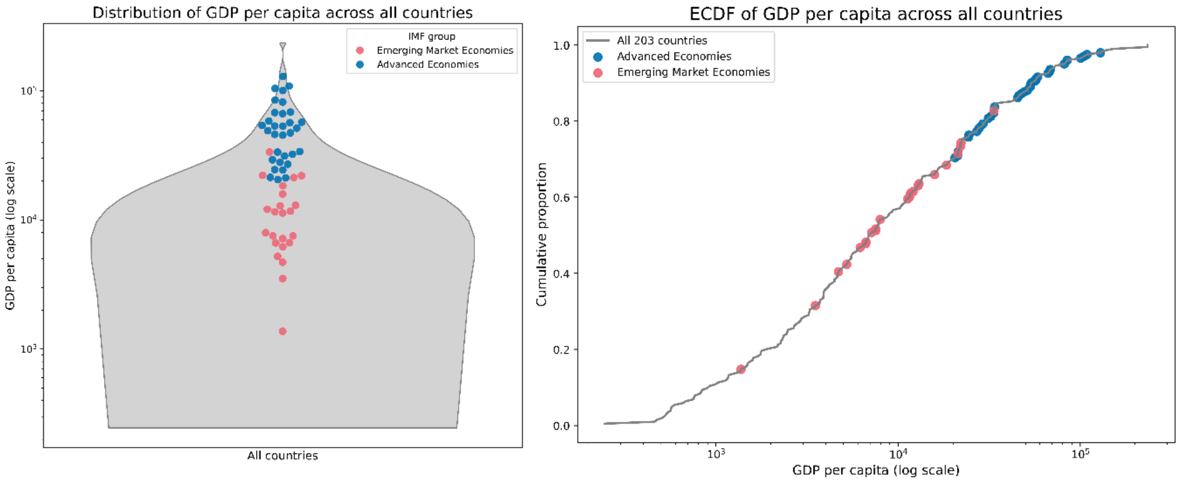

Table S2. List of Abbreviations in GHS Index.

Index	Abbreviation	Full domain name
1	Prevention	Prevention of the emergence or release of pathogens
2	Early detection	Early detection & reporting for epidemics of potential int'l concern
3	Response	Rapid response to and mitigation of the spread of an epidemic
4	Health system	Sufficient & robust health sector to treat the sick & protect health workers
5	Compliance	Commitments to improving national capacity, financing and adherence to norms
6	Risk environment	Overall risk environment and country vulnerability to biological threats

Table S3. Global Health Security Index scores by country (2021).

Country	Overall	1) Prevention	2) Early detection	3) Response	4) Health system	5) Compliance	6) Risk environment
Albania	45.0	42.0	40.0	38.1	47.4	52.1	50.6
Armenia	61.8	79.3	69.6	56.3	58.8	59.2	47.6
Australia	71.1	65.2	82.2	61.6	69.2	72.2	76.0
Austria	56.9	53.3	41.4	41.8	54.0	63.9	87.2
Azerbaijan	34.7	32.6	21.7	32.4	24.1	38.4	59.3
Belgium	59.3	54.2	52.9	46.4	64.2	61.1	77.2
Bosnia and Hercegovina	35.4	30.4	13.9	36.7	41.7	38.9	50.7
Brunei	43.5	30.1	44.7	44.0	34.9	41.5	65.9
Bulgaria	59.9	66.8	61.7	38.9	60.8	69.4	61.7
Canada	69.8	70.4	70.8	49.2	67.3	79.2	81.8
Croatia	48.8	47.7	37.8	31.0	51.4	59.7	65.0
Cyprus	41.9	44.1	25.0	34.0	32.3	52.8	62.9
Czech Republic	52.8	46.1	37.8	50.1	55.8	51.4	75.6
Denmark	64.4	64.3	64.6	51.8	64.5	61.1	79.9
Ecuador	50.8	50.5	51.5	42.0	56.7	53.1	50.9
Egypt	28.0	15.7	18.9	20.9	18.8	33.3	60.3
Estonia	55.5	42.5	41.3	56.2	49.4	66.7	76.9
Finland	70.9	58.2	67.5	70.7	68.7	77.8	82.6
France	61.9	59.4	45.7	47.7	70.4	65.3	82.9
Georgia	52.6	55.2	65.1	46.1	33.7	63.9	51.6
Germany	65.5	49.1	72.4	56.3	56.0	75.0	83.9
Greece	51.5	44.8	48.9	46.7	46.2	63.9	58.3
Hungary	54.4	49.4	38.1	50.1	54.6	62.5	71.7
Iceland	48.5	40.0	36.4	47.9	52.2	34.4	79.9
Ireland	55.3	52.9	50.4	41.4	51.7	55.6	79.9
Israel	47.2	41.6	46.7	44.4	55.2	30.9	64.2
Italy	51.9	47.2	49.7	43.2	40.2	65.3	65.9
Japan	60.5	43.1	71.1	59.5	51.6	66.7	70.9
Kazakhstan	46.1	54.9	29.2	36.5	34.6	58.7	62.9
Kyrgyz Republic	42.4	27.8	26.7	32.8	40.4	66.1	60.4
Latvia	61.9	51.6	77.1	51.2	60.6	59.7	71.3
Lithuania	59.5	38.2	64.3	58.7	59.9	62.5	73.3
Luxembourg	48.4	30.3	33.3	46.4	36.7	56.9	86.5
Malaysia	56.4	37.7	72.5	61.4	36.6	56.4	73.9
Malta	40.2	36.2	21.8	27.4	26.4	55.6	73.8

Mongolia	41.0	30.2	37.9	41.1	24.3	46.2	66.3
Montenegro	44.1	33.8	32.1	39.7	41.7	59.2	57.9
Netherlands	64.7	57.8	57.1	58.2	66.7	68.1	80.2
New Zealand	62.5	45.0	75.3	50.3	48.9	77.8	77.7
North Macedonia	42.2	35.7	37.1	35.2	38.3	47.4	59.7
Norway	60.2	53.8	46.3	57.5	45.0	69.4	89.0
Oman	39.1	35.4	33.5	31.7	28.6	41.5	64.2
Republic of Korea	65.4	48.8	73.8	65.0	62.5	69.4	73.1
Poland	55.7	43.5	42.5	53.3	52.7	72.2	70.1
Portugal	54.7	52.8	42.6	41.5	53.9	59.7	77.5
Romania	45.7	39.0	44.0	24.7	47.9	55.6	63.3
Russia	49.1	45.5	43.6	44.7	58.9	51.4	50.5
San Marino	32.9	17.4	21.4	36.6	28.6	18.8	74.7
Serbia	45.0	44.0	28.6	36.3	50.9	51.4	58.5
Singapore	57.4	46.8	61.1	61.3	47.3	48.6	79.5
Slovakia	54.4	51.3	37.1	43.7	62.7	59.7	72.2
Slovenia	67.8	65.7	70.8	59.9	72.8	63.9	73.4
South Africa	45.8	32.1	50.0	62.0	29.2	43.1	58.5
Spain	60.9	47.5	70.8	54.6	52.9	63.9	75.6
Switzerland	58.8	50.2	42.5	64.9	50.9	59.7	84.6
Thailand	68.2	59.7	91.5	67.3	64.7	68.9	57.2
Turkey	50.0	51.1	41.4	36.6	53.9	59.7	57.2
United Kingdom	67.2	63.5	70.8	64.8	68.3	62.5	73.0
United States of America	75.9	79.4	80.1	65.7	75.2	81.9	73.3

Table S4. Multivariable linear regression results for excess mortality in 2021 ($p < 0.05$(*), $p < 0.01$(**), and $p < 0.001$(***)).

Variable	Coefficient (β)	Standard error	95% CI	p-value	VIF
Intercept	0.8740	0.0800	[0.7163, 1.0344]	3.4e-15***	-
Prevention	0.0012	0.0016	[-0.0019, 0.0044]	0.4380	3.50
Early detection	-0.0024	0.0011	[-0.0047, -0.0002]	0.0367**	3.58
Response	0.0005	0.0016	[-0.0027, 0.0037]	0.7600	2.87
Health system	0.0002	0.0014	[-0.0027, 0.0030]	0.9040	3.26
Compliance	0.0001	0.0013	[-0.0025, 0.0028]	0.9260	2.30
Risk environment	-0.0096	0.0012	[-0.0121, -0.0072]	1.3e-10***	1.37
